# Activin signaling as an emerging target for therapeutic interventions

**DOI:** 10.1186/1478-811X-7-15

**Published:** 2009-06-18

**Authors:** Kunihiro Tsuchida, Masashi Nakatani, Keisuke Hitachi, Akiyoshi Uezumi, Yoshihide Sunada, Hiroshi Ageta, Kaoru Inokuchi

**Affiliations:** 1 Division for Therapies against Intractable Diseases, Institute for Comprehensive Medical Science (ICMS), Fujita Health University, Toyoake, Aichi 470-1192, Japan; 2 Division of Neurology, Department of Internal Medicine, Kawasaki Medical School, Kurashiki, Okayama 701-0192, Japan; 3 Mitsubishi Kagaku Institute of Life Sciences, MITILS, 11 Minamiooya, Machida, Tokyo 194-8511, Japan; 4 Japan Science and Technology Agency, CREST, Kawaguchi, Saitama 332-0012, Japan

## Abstract

After the initial discovery of activins as important regulators of reproduction, novel and diverse roles have been unraveled for them. Activins are expressed in various tissues and have a broad range of activities including the regulation of gonadal function, hormonal homeostasis, growth and differentiation of musculoskeletal tissues, regulation of growth and metastasis of cancer cells, proliferation and differentiation of embryonic stem cells, and even higher brain functions. Activins signal through a combination of type I and II transmembrane serine/threonine kinase receptors. Activin receptors are shared by multiple transforming growth factor-β (TGF-β) ligands such as myostatin, growth and differentiation factor-11 and nodal. Thus, although the activity of each ligand is distinct, they are also redundant, both physiologically and pathologically *in vivo*. Activin receptors activated by ligands phosphorylate the receptor-regulated Smads for TGF-β, Smad2 and 3. The Smad proteins then undergo multimerization with the co-mediator Smad4, and translocate into the nucleus to regulate the transcription of target genes in cooperation with nuclear cofactors. Signaling through receptors and Smads is controlled by multiple mechanisms including phosphorylation and other posttranslational modifications such as sumoylation, which affect potein localization, stability and transcriptional activity. Non-Smad signaling also plays an important role in activin signaling. Extracellularly, follistatin and related proteins bind to activins and related TGF-β ligands, and control the signaling and availability of ligands.

The functions of activins through activin receptors are pleiotrophic, cell type-specific and contextual, and they are involved in the etiology and pathogenesis of a variety of diseases. Accordingly, activin signaling may be a target for therapeutic interventions. In this review, we summarize the current knowledge on activin signaling and discuss the potential roles of this pathway as a molecular target of therapy for metabolic diseases, musculoskeletal disorders, cancers and neural damages.

## Signaling of activins and related growth factors through activin receptors

### Biosynthesis of activin and related growth factors

Activins belong to the transforming growth factor-β (TGF-β) family of growth and differentiation factors [1,2]. They form dimers composed of two inhibin β subunits. Four β subunits have been identified in mammals (βA, βB, βC and βE), whereas only a single inhibin α-subunit has been discovered so far. The βA and βB transcripts are found in nearly all tissues, whereas βC and βE subunits are expressed	predominantly in the liver. Both β and α subunits are synthesized as precursor polypeptides. After dimerization of the precursors, prodomains are cleaved by furin and/or related proprotein convertases in the endoplasmic reticulum and a mature dimeric polypeptide is released. Homodimers of inhibin βA or βB subunits, activin A and activin B, respectively, or heterodimeric activin AB exist in various tissues. Inhibins, heterodimeric proteins composed of an α-subunit linked to β-subunits by disulfide bonds, act as activin antagonists. In the case of myostatin, another TGF-β family protein related to activins, cleavage and maturation of the ligand may occur extracellularly in a tissue-specific manner [3].

### Activin receptors

Activin signals are transmitted through two types of transmembrane serine/threonine kinase receptors, type I and type II activin receptors in target cells [1,4]. Activin receptors are prototypes of single-pass transmembrane serine/threonine kinases. Intriguingly, activin receptors are shared by other TGF-β family proteins, such as myostatin, growth and differentiation factor 11 (GDF11) and nodal. Therefore, several activities of these ligands are redundant with those of activins. Myostatin has been characterized as a skeletal muscle-specific cytokine regulating skeletal muscle mass [5]. GDF11 is structurally similar to myostatin, and is involved in neurogenesis in the spinal cord and olfactory bulb [6]. GDF11 also regulates kidney development and endocrine pancreas development [7,8]. Nodal is a central player in patterning the early embryo during the induction of mesoderm and endoderm [9], and acts as an authentic mesoderm inducer in mammalian species. Some of these activities are shared with activins.

**Figure 1:**
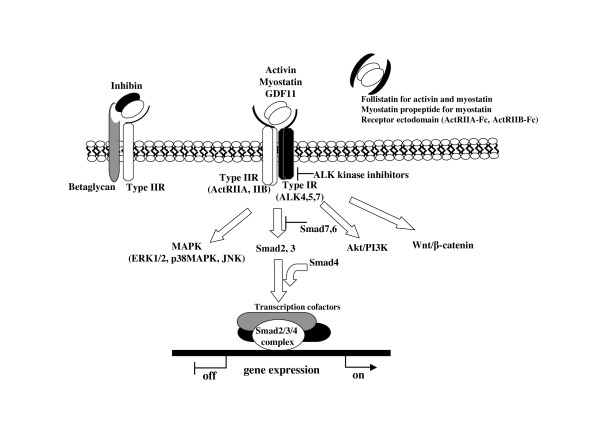
**Signal transduction through activin receptors**. Activin, myostatin and GDF11 signal through type II and type I serine/threonine kinase receptors. Type IIR is the principal ligand binding receptors, and ligand/typeIIR complexes recruit and associate with type IR. Type IR is phosphorylated and activated by type IIR kinase. Smad2 and 3, activin/TGF-β specific Smads, are phosphorylated by activated type IR. In the nucleus, Smad2/3/4 complexes regulate gene expression with additional transcriptional cofactors. Smad-independent pathway such as MAPK is also activated downstream of activin receptors. Inhibin acts antagonistic to activin by forming high affinity complexes with ActRII and betaglycan. Follistatin, myostatin propeptide and receptor ectodomain inhibit the activities of activin and related factors in the extracellular space to prevent ligand/receptor interaction. Chemical type IR kinase inhibitors act in the cell to disrupt receptor/intracellular signaling.

Activin type II receptor, ACVR2 or ActRIIA, has been identified and characterized as a transmembrane serine/threonine kinase for activin A [10]. A second activin type II receptor, ACVR2B or ActRIIB, has also been identified [4]. In addition, TGF-β type II receptor, BMP type II receptor and Müllerian duct inhibiting substance type II receptor specific to each ligand have been characterized [2]. To date, seven type I receptors, activin receptor-like kinases 1 to 7 (ALK1-7), have been characterized for the TGF-β family [11]. Like type II receptors, type I receptors possess a serine/threonine kinase domain. However, different from type II receptors, type I receptors have a unique GS domain near the intracellular juxtamembrane regions preceding the kinase domain. The amino acid sequences of L45 loops of type I receptors located between the kinase subdomains IV and V are responsible for the preference of Smad proteins and determine the specificity between the activin/TGF-β subgroup (ALK4, 5, 7) and BMP subgroup (ALK1, 2, 3, 6) [2,11]. ALK4 is known as activin type IB receptor, ACVR1B or ActRIB, whereas ALK7 is known as activin type IC receptor, ACVR1C. ALK4 and ALK7 are type I receptors for activins and nodal, and ALK4 and ALK5 are receptors for myostatin and GDF11 (Table S1; additional file 1) [1,2]. Once activins bind to ActRIIA or ActRIIB, type I receptors are recruited to the ligand/ActRII complex, and the GS domains of type I receptors become phosphorylated by ActRII kinases. Activin/TGF-β-specific Smad, Smad2 and Smad 3, are phosphorylated by activated type I receptors (Figure 1). In the case of nodal, the co-receptor Cripto and related factors are required for the complete activation [9]. Cripto facilitates nodal signaling by binding to both nodal and activin receptors. Interestingly, Cripto may also act as an inhibitory factor for activin signaling when overexpressed [12](Table S1; additional file 1).

A pseudo-receptor BMP and activin membrane-bound inhibitor, BAMBI, has been identified [13]. BAMBI interacts with multiple type I receptors for TGF-β family ligands and inhibits the formation of the active receptor signaling complex. Thus, BAMBI serves as an endogenous dominant negative receptor [13]. BAMBI is characterized as a β-catenin target in colorectal tumors [14].

### Regulation of activin receptors

Regulatory proteins for activin receptors control the signaling activity of activins and related growth factors. A FYVE domain-containing protein, the Smad anchor for receptor activation (SARA), interacts with both the type I receptor and Smads.

Complex formation of activin receptors with SARA and Smad in EEA-1 positive early endosomes may be an essential step for efficient activin/TGF-β signaling [15,16]. Activin type II receptors (ActRIIA and ActRIIB) have consensus amino acids for PSD-95/Discs-large/ZO-1 (PDZ) protein interaction at their COOH-terminus [1]. This characteristic is unique among receptors of the TGF-β family [17]. Activin-receptor interacting proteins (ARIPs), which have PDZ domains, associate with the COOH-terminus of ActRIIs and regulate activin signaling. ARIP1 has multiple WW and PDZ domains for protein-protein interactions, and regulates the localization of activin receptors and negatively controls signaling [17]. Intriguingly, ARIP-1 acts as a scaffold for N-methyl-D-aspartate (NMDA) receptor activation in hippocampal neurons, and is also known as synaptic scaffolding protein, S-SCAM [18]. A recent study showed that activin induces long-lasting NMDA receptor activation by ARIP1 in hippocampal neurons [19]. ARIP2 is a small protein that has one PDZ domain [20]. Several ARIP2 splicing isoforms exist, and, depending on the isoform, ARIP2 either augments or inhibits activin signaling [21]. Gene trapping analysis identified the RasGAP-binding protein Dok-1, which acts downstream of receptor tyrosine kinases as an essential adapter molecule for activin-induced apoptotic signaling in B cells. Dok-1 interacts simultaneously with activin receptors and Smads. Stimulation by activin induces association of Dok-1 and Smad3 [22].

Posttranslational modification of the activin/TGF-β receptor is an additional important mechanism for the regulation of receptor activation. The ubiquitin-proteasome pathway tightly regulates TGF-β family signaling. HECT-type E3 ubiquitin ligases, Smad ubiquitin regulatory factor 1 (Smurf1) and Smurf2 have been implicated in Smad degradation. Smurf1 and Smurf2 bind to TGF-β family receptors via the inhibitory Smads, Smad6 and Smad7, to induce their ubiquitin-dependent degradation [23]. In addition, TGF-β type I receptor is sumoylated in response to ligand stimulation. Posttranslational receptor sumoylation, the covalent attachment of a small ubiquitin-like modifier (SUMO) is required for the kinase activities of both the TGF-β type I and type II receptors, and enhances receptor function by facilitating the recruitment and phosphorylation of Smad3 [24].

### Regulation of activin signaling through Smads

Smad signaling in the cytoplasm and the nucleus is under tight control. Smads consist of an NH_2_-terminal MH1 and a COOH-terminal MH2 domain. The L45 loop of type I receptors directly interacts with the MH2 domain of receptor-regulated Smad (R-Smad), and determines Smad specificity [2]. Type I receptors phosphorylate Smads at their COOH-terminal two serine residues. Smad2 and 3, R-Smads for activin and TGF-β undergo constant shuttling between the cytoplasm and nucleus, and the activation of R-Smads triggers nuclear accumulation [2]. PPM1A may act as a Smad COOH-terminal phosphatase [25]. Linker regions between MH1 and MH2 domains of Smads are phosphorylated by mitogen-activated protein kinase (MAPK). This phosphorylation enhances the binding of ubiquitin ligase to Smad, resulting in polyubiquitination and degradation [26].

Smads have intrinsic DNA-binding activity [2]. However, to fully activate target genes, Smad physically associates with a diverse set of DNA-binding cofactors such as CBP/p300, TGIF, c-Ski and Evi-1 [11]. This characteristic determines the cell type-specific transcription and complexity of activin/TGF-β signaling. A number of transcription factors including forkhead proteins, bHLH family, AP1 family, homeodomain protein family and nuclear receptors act as Smad-interacting transcription factors [2]. Once activated, Smad complexes recruit additional transcriptional activators or repressors to regulate target genes (Figure 1).

Negative feedback regulation by the inhibitory Smads, Smad6 and Smad7 is an important shutoff system for signaling by the TGF-β family including activins [2,11].

### Smad-independent activin signaling and receptor crosstalk

In addition to the canonical Smad pathway, activin signaling through activin receptors regulates other intracellular pathways. p38 MAPK, ERK1/2 and JNK are activated by activin in a cell type-specific manner [27,28]. For example, activin synergizes with basic fibroblast growth factor to activate tyrosine hydroxylase expression via the ERK1/2 pathway [27]. Activin negatively regulates the pituitary transcription factor Pit-1 through p38 MAPK-dependent and Smad-independent pathways [28]. Independently of Smad4, ActRIB/Smad2 acts as a co-activator of the canonical Wnt signaling pathway. Upon activation, Smad2 physically interacts with Tcf4, β-catenin and the co-activator p300 to enhance transcriptional activity of β-catenin/Tcf4 through the histone acetyltransferase activity of p300 [29]. Transactivation by Smad2 is independent of the Smad binding element. Furthermore, recent characterization revealed that TGF-β stimulates phosphorylation of BMP-specific Smad1 independently of BMP receptors [30-32]. Smad-independent activin signaling and receptor crosstalk increase the complexity of activin/TGF-β signaling.

### Ligand binding proteins

Extracellular activin-binding proteins control activin signaling [1]. Follistatin (FST) is a prototype of activin-binding proteins. FST is a cysteine-rich single chain glycoprotein that does not possess sequence similarity to the TGF-β family [33]. Structural analysis of FST with activin showed that two FST molecules encircle activin, and neutralize the ligand by burying one-third of its residues and both type II and type I receptor binding sites [34-36] (Figure 1). FST not only binds and inhibits activins, but also binds and neutralizes the actions of myostatin and GDF11 [1,37]. Mice with a disrupted follistatin gene have musculoskeletal and cutaneous abnormalities, reflecting the abnormal signaling of activins, myostatin and GDF11 [38]. The follistatin-related gene, FLRG, is a follistatin domain-containing protein structurally similar to FST [39,40]. Whereas FST has three follistatin domains, FLRG has only two. Like FST, FLRG binds and neutralizes activins, myostatin and GDF11 [37,39]. Proteomics analyses indicate that FLRG associates with myostatin in sera [37]. Although functionally redundant, expression and transcriptional regulation of FST and FLRG are different [39-41]. FLRG gene deleted mice show dysregulated glucose metabolism and fat homeostasis [42](see below).

## Biological activities and roles of activin signaling as a target of therapeutic interventions

After the purification and identification of activins as regulators of follicle-stimulating hormone secretion from the anterior pituitary, important roles of activins in the hypothalamus-pituitary-gonadal axis have been described [1]. However, activin activity is not limited to reproductive tissues. Activins and related factors have pleiotropic actions in extragonadal tissues. In this section, we focus on selective actions of activins and related growth factors from a therapeutic point of view.

### Activins and their regulators in metabolic disorders

Activin signaling is required for proper development of the endocrine and exocrine pancreas, and dysregulation of the activin signaling pathway contributes to the genesis of metabolic diseases. In human embryonic stem cells, activin B mediates the induction of homeoprotein Pdx1, a key regulator of endocrine pancreas development [43]. ActRIIA mutant mice show hypoplasia of the pancreas and develop diabetes [44]. ActRIIB and Smad2 activity use the same signaling pathway to regulate pancreas islet formation [45]. ALK7, a type I receptor for activin B, activin AB and nodal, is expressed abundantly in pancreatic β cells and adipose tissues, and regulates insulin biosynthesis and secretion [46-48]. Recent characterization revealed that ALK7 transmits signals of GDF3, another TGF-β family member [49,50]. GDF3, ALK7 and co-receptor Cripto are all expressed in adipose tissues, and Gdf3(-/-) null mice and ALK7(-/-) null mice showed reduced fat accumulation and resistance to diet-induced obesity [49,50].

The expression of activin receptors, myostatin and their binding protein FLRG can be modulated in adipose tissue and skeletal muscle by chronic obesity. In subcutaneous and visceral fats, myostatin and ActRIIB mRNA levels in ob/ob mice are 50- to 100-fold higher than that in wild-type mice [51]. By contrast, FLRG mRNA levels are increased in subcutaneous fat, but decreased in visceral fat of ob/ob mice compared to wild-type mice [51]. In humans, myostatin was shown to increase in skeletal muscle and plasma of obese and insulin resistant women [52].

FLRG gene disrupted mice showed an increased pancreatic islet number and size, β cell hyperplasia, decreased visceral fat mass, improved glucose tolerance, and enhanced insulin sensitivity. This phenotype is caused through increased signaling by activin or myostatin in a tissue-specific manner [42].

### Myostatin and activin in muscular diseases

Myostatin, the skeletal muscle specific member of the TGF-β family, restricts muscle growth and determines skeletal muscle mass [5]. Myostatin signals through activin type I receptors (Alk4 and 5) and type II receptors [5]. Mice with a targeted deletion of the myostatin gene have a 25–30% increased muscle mass resulting from hypertrophy and hyperplasia [53]. Double muscling phenotypes upon inactivation of the myostatin gene have been observed in cattle, sheep, race dogs, fish and even in humans [54-59]. Myostatin is regarded as a good drug target since therapeutics that stimulate skeletal muscle growth may be useful for muscle-wasting conditions such as muscular dystrophy, sarcopenia and cachexia. Whereas activins and TGF-β function in almost every cell type, myostatin specifically affects skeletal muscle growth. Thus, targeting myostatin is a rational therapeutic strategy to increase skeletal muscle mass. Several myostatin inhibitors such as monoclonal antibodies and myostatin propeptide, as well as FST and its derivatives are promising candidates for the treatment of muscle wasting disorders [60-67] (Table S2; Additional file 2). Skeletal muscle fibrosis is also ameliorated by myostatin inhibition [68]. The effectiveness of myostatin inhibition has been studied using various muscular dystrophy animal models. Monoclonal antibody-mediated myostatin blockade results in an increase of muscle mass and absolute muscle strength in *mdx *mice, an animal model of Duchenne-type muscular dystrophy [60]. Muscles in *mdx *mice with myostatin inhibition showed less fibrosis, reduced fatty remodeling and an improved regeneration process [61]. Myostatin circulates in the serum in a latent form complexed with multiple binding proteins. NH_2_-terminal myostatin propeptide is a major myostatin-binding protein and non-covalently associates with myostatin [5,37]. Myostatin propeptide, stabilized by fusion to IgG-Fc, has been shown to be effective in ameliorating dystrophic pathophysiology [62]. Muscle atrophy caused in mutant caveolin-3 transgenic mice, a model of limb-girdle muscular dystrophy (LGMD) 1C, was reduced dramatically by crossing these mice with myostatin propeptide transgenic mice [63]. In calpain 3-deficient LGMD2A model mice, both muscle mass and muscle force were recovered upon gene therapy using myostatin propeptide [64]. Myostatin blockage at an early stage in a model of δ-sarcoglycan-deficient muscular dystrophy was effective in reducing muscle loss and fibrosis, and in improving regeneration [65]. It is of note that the elimination of myostatin did not suppress the phenotype of a laminin-α2-deficient mice, but increased postnatal lethality due to fat loss [69]. Soluble forms of an extracellular domain of ActRIIB fused with IgG-Fc may block myostatin effectively *in vivo*, and have strong muscle mass increasing activities [70]. In addition to myostatin, activin and GDF11 are recognized by soluble forms of ActRIIB [71]. FST and FST-derived myostatin inhibitors are also effective for increasing muscle mass and ameliorating muscular dystrophy [66,67]. It is worth noting that neurogenic muscle atrophy caused by amyotrophic lateral sclerosis and spinal muscular atrophy may be ameliorated by myostatin inhibition either by myostatin antibody or follistatin [72,73].

The expression of activin, myostatin, TGF-β, activin receptors, and FST in cardiac muscle is also deregulated in pathological conditions such as cardiac failure and cardiomyopathy [74,75]. However, in contrast to the observations in skeletal muscle, myostatin does not counteract cardiac hypertrophy or fibrosis [75].

### Roles of activin and BMP signaling in osteoporosis and bone formation

Although both BMP and activin regulate bone formation, their modes of action are distinct. BMPs are potent inducers of osteoblast differentiation. Activins are expressed abundantly in bone tissues, and regulate bone formation by controlling both osteoblast and osteoclast functions. Different from the activity of BMP, activins enhance the receptor activator of NF-κB ligand (RANKL)-mediated osteoclast differentiation, and act as commitment factors for osteoclastogenesis [76]. Both antiresorptive and anabolic drugs are useful for the treatment of osteoporosis [77]. Bisphosphonates, selective estrogen-receptor modulators and estrogen are currently available antiresorptive drugs, whereas recombinant human parathyroid hormone is an anabolic drug. Intriguingly, the extracellular domain of ActRIIA stabilized by fusion to IgG-Fc increases bone mass and strength by activin inhibition, and is a novel promising agent for osteoporosis in early human trials [77,78] (Table S2; Additional file 2).

As mentioned above, the extracellular domain of ActRIIB fused to IgG-Fc increases muscle mass. Thus, two activin type II receptor decoys have different clinical uses. Consistent with the activity of activin in bone formation, inhibin A, an activin antagonist, works as an endocrine stimulator of bone mass *in vivo *by increasing osteoblastogenesis [79]. Inhibin antagonizes activin by forming a complex of ActRIIs and betaglycan [2,4](Figure 1).

Fibrodysplasia ossificans progressive (FOP), a genetic disorder of progressive heterotypic ossification, is caused by missense mutations in ACVR1A (ALK2), a BMP type I receptor, which increase BMP signaling [80]. A recurrent activating mutation in the juxtamembrane GS domain of ACVR1A was reported in sporadic and familial cases of classic FOP [80]. Thus, the activin and BMP pathway are therapeutic targets for the treatment of low bone mass.

### Roles of activins and related growth factors in cancer

Inhibition of cancer cell growth is one of the activities of activins in the early phase of cancer development. Facilitating activin signaling either by Cripto silencing or FLRG silencing inhibits human breast cancer cell growth [81,82](Table S2; Additional file 2). Mutations in several genes involved in the activin signaling pathway have been characterized in cancers. Two 8-bp polyadenine tracts of the ACVR2 gene were targets for frameshift mutations in gastrointestinal cancers with microsatellite instability [83]. Somatic ACVR1B gene mutations have been found in pancreatic carcinoma [84] and Smad2 and Smad4 are mutated in colorectal and pancreatic carcinomas [85]. Thus, dysregulation of activin receptors and activin/TGF-β Smads is directly involved in carcinogenesis.

Interestingly, inhibin-deficient mice develop gonadal sex cord-stromal tumors [86]. They develop adrenal cortical tumors when gonadectomized. Therefore, inhibins act as secreted tumor suppressors in gonads and adrenal glands. Supraphysiological levels of activins in inhibin-deficient mice are responsible for the development of tumors. Overproduction of activins was observed in a cachexia-like wasting syndrome that includes hepatocellular necrosis and metastasis [86-88]. Thus, the actions of activin in tumor development are highly context-dependent.

Myofibroblasts present in tumor stroma facilitate tumor development and invasion [2]. TGF-β and activin stimulate the differentiation of myofibroblasts from mesenchymal progenitors, suggesting the facilitation of invasive properties of cancers.

Regarding metastasis, inhibition of activin and/or TGF-β suppresses experimental metastasis to multiple organs including lung, liver and bone [89,90](Table S2; Additional file 2). Chemical inhibitors for type I receptor kinases for activin/TGF-β (ALK4, 5 and 7) are promising cancer therapies [89,91]. They may offer an option for preventing tumor angiogenesis, the motility of cancer cells, fibrosis and metastasis [92].

TGF-β and TGF-β type I receptor are upregulated at the tumor-bone interface and modulate RANKL-dependent osteolysis, and TGF-β inhibition reduces mammary tumor-induced osteolysis [93]. Since activin works as a cofactor for RANKL, similar to TGF-β, activin may modulate osteoclastogenesis in the tumor-bone  interaction.

TGF-β produced by cancer cells has immunosuppressive effects, resulting in the evasion of cancers from destruction by the immune system. A novel TGF-β kinase inhibitor reverses this effect, inhibits cell growth and enhances the immunogenicity of cancer cells [94]. Whether activins also act as regulators in immunosuppression in cancers has not yet been determined.

### Activities of activins in the brain

Activins and activin receptors are expressed highly in the central nervous system and have crucial roles in neuronal development [95,96]. However, compared with classical neurotrophic factors, our knowledge about the functions of activins in the brain is limited. Importantly, the expression of inhibin βA mRNA, which encodes activin A, is induced by excitatory synaptic input [97,98]. It is induced in granule cell neurons of the hippocampus by high-frequency synaptic stimuli that produce long term potentiation (LTP). This induction is NMDA receptor-dependent [97,98]. Activin increases the number of synaptic contacts by modulating actin dynamics in the spine of the neurons, which may be responsible for the establishment of LTP [99]. This modulation is mediated by the classical MAP kinase cascades via Erk1/2 [99]. Similarly, inhibin βA mRNA is transiently induced in dentate gyrus neurons through NMDA receptor activation after unilateral mechanical brain injury by saline injection [100]. Inhibin βA mRNA is also induced during amygdala kindling, and accurately marks excitatory neurons with synaptic alterations from seizures [101].

Accumulating evidence indicates that activin also has neurotrophic and neuroprotective effects on selective neurons [102]. Treatment with recombinant activin following ischemic injury rescues neurons from damage [103]. Overexcited neurons are protected by the neurotrophic effect of basic fibroblast growth factor, which depends on the induction of activin A [104] (Table S2; Additional file 2). It is also of note that activin and fibroblast growth factor act in synergy in dopaminergic neurons [27].

Neuronal-specific transgenic approaches using the αCaMKII promoter revealed further functions of activins [105,106]. Hippocampal neurons in αCaMKII promoter-driven dominant negative ActRIB transgenic mice were more vulnerable to kainate injection [105]. These mice also showed a reduced NMDA current with an impaired LTP. Reciprocally, activin potentiates NMDA receptor-mediated signaling by forming complexes with activin receptors, NMDA receptors and Fyn on postsynaptic scaffolding proteins [19]. Interestingly, activins tune pre- and postsynaptic GABAergic transmission affecting anxiety [107]. αCaMKII promoter-driven activin and FST transgenic mice are affected in their anxiety-related behavior by modulation of their postnatal neurogenesis in the subgranular zone of the dentate gyrus in the hippocampus [106]. Infusion of activin into the dentate gyrus of the hippocampus produces an antidepressant-like effect in the forced swim test. Conversely, antidepressants such as fluoxetine and desipramine increase Smad2 phosphorylation [108]. These data suggest that the activin signaling pathway may be a novel target for neuroprotection and psychopharmacological therapy.

### Role of activins in embryonic stem cells

Activin A is a potent mesoderm inducer in *Xenopus *embryos, and numerous tissues can be differentiated from *Xenopus *animal cap cells and embryonic stem cells [109]. A sophisticated strategy to differentiate mouse embryonic stem cells into insulin-producing cells or other cell types by activin has been developed [110,111]. Intriguingly, activin signaling is indispensable to maintain self-renewal and the stemness of human embryonic stem cells [111]. Activin signaling sustains the expression of pluripotency-associated genes such as nanog and inhibits BMP signaling, which promotes self-renewal in human embryonic stem cells [112].

## Conclusion

### Activin signaling as a target for therapeutic intervention

Although activins were first discovered as powerful factors to stimulate follicle-stimulating hormone production from the anterior pituitary, activins act on almost all cell types and have diverse roles. Furthermore, activin receptors are shared by other TGF-β family members such as myostatin, GDF11, nodal and a subset of BMPs. The TGF-β family members are key regulators of myogenesis, neurogenesis and organogenesis, left-right asymmetry and bone formation. Actions of activins through activin receptors and Smads are pleiotropic and context-dependent, and alterations in signaling through activin receptors are the cause of a variety of disorders. In this review, we focused on recently characterized aspects of activin signaling in relationship to metabolic diseases, musculoskeletal diseases, cancers and neuroprotection.

Various strategies have been designed for the inhibition of activin signaling through receptors. Soluble forms of the extracellular domains of activin receptors, FST and related ligand binding proteins, chemical kinase inhibitors for activin receptors, and siRNAs either for ligand or signaling molecules interfere with activin signaling. Intriguingly, histone deacetylase inhibitors or nitric oxide have been demonstrated to inhibit the progression of muscular dystrophy in a mouse model by transcriptional activation of FST [113,114].

In muscle wasting disorders, the inhibition of myostatin is a possible therapeutic strategy. Soluble ActRIIB-Fc, FST and its derivatives, myostatin propeptide, monoclonal myostatin antibodies and myostatin siRNA are myostatin inhibitors that have been shown to be beneficial for preventing muscle loss. Cachexia from cancers and neurogenic muscle atrophy are also targets for myostatin inhibition [72,73,115](Table S2; Additional file 2).

In cancers, activins have multiple roles such as regulation of cancer cell growth, promotion of organ-specific cancer progression and metastasis. Soluble ActRIIA-Fc is a novel promising drug for osteoporosis, cancer-related bone loss and cachexia [77,78,88]. Activin also has neuroprotective functions, and the augmentation of activins may have favorable protective effects on neurons (Table S2; Additional file 2).

Although targeting activin and related factors may become part of future therapies, given the complexity of their action, some side-effects of such therapies are certainly possible. The dysregulation of activin may affect functions of gonads and adipose tissues [4,42]. It is also possible that activation or targeting activin/TGF-β may in some contexts cause uncontrollable tumor growth or detrimental cellular apoptosis [22,86].

Once promising proteins or chemicals targeting activin signaling are discovered, methods of the drug delivery system are important issues for effective treatment. The stabilization of peptides by fusion with IgG-Fc or other stable proteins is a strategy for targeting activin signaling. Delivery of genes by adeno-associated viral vectors is also potentially promising [64,116]. Finally, nanoparticles such as liposomes and atellocollagen are efficient delivery vehicles for siRNA and proteins [117], and may be useful in delivering agents that target activin signaling.

In summary, therapeutic interventions targeted to signaling through activin receptors may provide novel strategies for the development of effective treatments against a variety of diseases.

## Abbreviations

TGF-β: transforming growth factor-β; GDF11: growth and differentiation factor 11; ACVR2 or ActRIIA: activin type II receptor; ACVR2B or ActRIIB: activin type IIB receptor; BMP: bone morphogenetic protein; ALK: activin receptor-like kinase; ACVR1B or ActRIB: activin type IB receptor; ACVR1C: activin type IC receptor; BAMBI: BMP and activin membrane-bound inhibitor; PDZ: PSD-95/Discs-large/ZO-1; ARIP: activin receptor interacting protein; NMDA: N-methyl-D-aspartate; MAPK: mitogen-activated protein kinase; FST: follistatin; FLRG: follistatin-related gene; LGMD: limb-girdle muscular dystrophy; RANKL: receptor activator of NF-κB ligand; FOP: fibrodysplasia ossificans progressive; ACVR1A: activin type IA receptor; LTP: long term potentiation; αCAMKII: α calmodulin kinase II.

## Competing interests

The authors declare that they have no competing interests.

## Authors' contributions

MN participated in the analysis of MSTN/activin signaling and muscle diseases. KH participated in the analysis of growth factor signaling and the interaction of growth factors. AU participated in the analysis of skeletal muscle differentiation. YS participated in therapy for muscular dystrophy. HA and KI participated in the functions of activins in the central nervous system. KT conceived of the study, and participated in its coordination. All authors approved the manuscript.

## Supplementary Material

Additional file 1 **Table S1. Ligand/receptor combination for activin and related factors**. The table provided represents the ligand/receptor combination for activins, inhibins, myostatin, GDF11 and nodal. Click here for file

Additional file 2 **Table S2. Activin signaling as a target for therapeutic interventions**. The table provided represents activin signaling as a target for therapeutic interventions and lists the disease, therapeutic strategy, methods and references. Click here for file
