# Supplementary material for: Activin signaling as an emerging target for therapeutic interventions
Source: Cell Commun Signal. 2009 Jun 18;7:15. doi: 10.1186/1478-811X-7-15 (PMC2713245; doi:10.1186/1478-811X-7-15)
Supplement: Additional file 1 — Table S1. Ligand/receptor combination for activin and related factors. The table provided represents the ligand/receptor combination for activins, inhibins, myostatin, GDF11 and nodal. [file 1478-811X-7-15-S1.pdf]

Table 1. Ligand/receptor combination for activin and related factors

Ligand	Type II Receptor	Type I Receptor	Coreceptor	Smad
Activin A	ActRIIA, IIB	ALK4, (7)	-	Smad 2,3 with Smad4
Activin B	ActRIIA, IIB	ALK7, (4)	-	
Activin AB	ActRIIA, IIB	ALK4, 7	-	
Inhibin A, B	ActRIIA, IIB	-	Betaglycan	
Myostatin	ActRIIB, (IIA)	ALK5, (4)	-	Smad 2,3 with Smad4
GDF11	ActRIIB, IIA	ALK4, 5	-	
Nodal	ActRIIB, IIA	ALK4, 7	Cripto	Smad 2,3 with Smad4

Note: weak interaction of ActRIIA and ALK4 with myostatin.
weak interaction of ALK7 with activin A.
weak interaction of ALK4 with activin B.
