# Supplementary material for: Activin signaling as an emerging target for therapeutic interventions
Source: Cell Commun Signal. 2009 Jun 18;7:15. doi: 10.1186/1478-811X-7-15 (PMC2713245; doi:10.1186/1478-811X-7-15)
Supplement: Additional file 2 — Table S2. Activin signaling as a target for therapeutic interventions. The table provided represents activin signaling as a target for therapeutic interventions and lists the disease, therapeutic strategy, methods and references. [file 1478-811X-7-15-S2.pdf]

Table 2. Activin signaling as a target for therapeutic interventions

Disease	Therapeutic strategy	Methods	Ref
Muscular dystrophy<br>Muscle atrophy	Increase of muscle mass by myostatin inhibition	Monoclonal MSTN Ab<br>MSTN propeptide<br>Soluble ActRIIB-Fc<br>Follistatin and its derivatives<br>HDAC inh<br>MSTN siRNA	[60, 65, 72]<br>[62, 63, 64]<br>[63, 70]<br>[66, 67, 73, 116]<br>[113]<br>[117]
Osteoporosis	Increase of bone mass by activin inhibition	Soluble ActRIIA-Fc<br>Inhibin A	[78]<br>[79]
Cancer<br>tumor growth	Suppression by activin activation	Cripto silencing<br>FLRG silencing	[81]<br>[82]
cachexia	Activin inhibition<br>Myostatin inhibition	Soluble ActRIIA-Fc<br>Follistatin, MSTN propeptide	[88]<br>[115]
metastasis	Activin inhibition	Follistatin	[90]
angiogenesis and motility	Suppression by TGF-β/activin inhibition	ALK4, 5, 7 kinase inhibitors	[89, 91, 92, 94]
Neuron damage<br>Depression	Recombinant Activin A application	Activin A	[102, 103, 104, 108]

Abbreviations: MSTN, myostatin; HDAC inh, histone deacetylase inhibitor
